# Predicting Outcome in Adult Patients With Congenital Heart Disease: A Systematic Review and Meta-Analysis on the Predictive Value of NT-proBNP and High-Sensitive Troponin T

**DOI:** 10.1155/ijvm/1210312

**Published:** 2025-07-03

**Authors:** Sara Rashki Ghalenoo, Zohreh Mahmoodi

**Affiliations:** Department of Cardiovascular Diseases, Faculty of Medicine, Mashhad University of Medical Sciences, Mashhad, Iran

**Keywords:** congenital heart disease, high-sensitive troponin t, N-terminal pro-B-type natriuretic peptide

## Abstract

**Objective:** This study is aimed at evaluating the predictive value of high-sensitive cardiac troponin T (hs-TnT), and N-terminal probrain natriuretic peptide (NT-proBNP), for cardiovascular events and/or survival in stable adult congenital heart disease (ACHD) patients.

**Methods:** A systematic review along with a meta-analysis was done on studies from 2014 to 2024 that examined hs-TnT, NT-proBNP, and their association with cardiac events and/or mortality in adult patients with congenital heart disease. A comprehensive search was conducted across major databases, and studies reporting biomarker levels and relevant outcomes were included. Data on study characteristics and hazard ratios (HRs) were extracted, and pooled estimates were calculated using random-effects meta-analysis, with heterogeneity assessed through the *I*^2^ statistic. STATA software was used for data analysis.

**Results:** A total of five studies, consisting of 1294 adult congenital heart disease (ACHD) patients, were included in this meta-analysis. Elevated NT-proBNP levels were significantly associated with an increased risk of mortality or cardiac events (HR: 2.13; 95% CI: 1.84–2.42), which remained significant after adjustment for confounding factors (adjusted HR: 2.34; 95% CI: 1.55–3.13). Elevated hs-TnT levels were also associated with a higher risk of adverse outcomes (HR: 1.57; 95% CI: 1.36–1.78), with the association remaining significant after adjustment (adjusted HR: 2.65; 95% CI: 1.22–5.76). Sensitivity analysis excluding a study with a lower hs-TnT cut-off further strengthened the association (adjusted HR: 3.03; 95% CI: 0.86–5.21) and reduced heterogeneity.

**Conclusion:** In conclusion, this meta-analysis shows the prognostic value of both NT-proBNP and hs-TnT in adults with congenital heart disease. Each of these markers offered a distinct but complementary clinical insight. Although methodological differences of the included studies limit direct comparison, our systematic review supports the potential value of incorporating both biomarkers into routine risk assessment.

## 1. Introduction

Congenital heart disease (CHD) refers to significant structural abnormalities of the heart and/or major thoracic vessels that arise during the embryonic stage [1]. Currently, CHD is the most common congenital anomaly globally and is a leading cause of perinatal mortality, with a birth prevalence of approximately 10% worldwide [2–4].

Over the past five decades, surgical interventions for children with congenital heart disorders have seen significant advancements. As a result, more than 90% of these children now live to reach adulthood [5–7]. As a result the number of these adults with CHD continues to grow and is expected to reach a peak around 2050. However, many of these individuals still have residual cardiac abnormalities, which increase the risk of complications such as arrhythmias, heart failure, and premature mortality [8–10].

Given this, there is a critical need to develop prognostic tools to assess the risk of adverse events in the long-term among adult CHD (ACHD) patients. Such tools would facilitate early detection, prevention, and timely intervention, ultimately improving survival rates and quality of life for ACHD patients.

Blood biomarkers are crucial in diagnosing and managing congestive heart failure within cardiology. Several biomarkers reflect the different pathophysiological aspects and are linked to severe complications in ACHD [11].

A marker which is indicative of myocardial stress is called N-terminal probrain natriuretic peptide (NT-proBNP). This marker is a well-known predictor of negative outcomes both in general cardiology and ACHD, where rising levels during follow-up correlate with increased heart failure risk and mortality [12, 13]. On the other hand, high-sensitive cardiac troponin T (hs-TnT) is an indicator of damage to cardiomyocytes and has shown notably elevated levels in ACHD patients demonstrating associations with heart failure symptoms [14].

Although several studies have investigated the prognostic value of either NT-proBNP or hs-TnT in patients with CHD [15–18], few have evaluated both biomarkers concurrently within the same patient population [19–25]. Moreover, the available studies are often limited by small sample sizes, single-center designs, and heterogeneous methodologies. As a result, the comparative prognostic performance of these biomarkers, particularly in stable ACHD patients, remains unclear. To address this gap, we conducted a comprehensive systematic review and meta-analysis to synthesize the existing evidence, compare the prognostic value of NT-proBNP and hs-TnT within the same patient populations, and offer clinicians' clearer insights into how these biomarkers may guide risk stratification and clinical decision-making in adults with CHD.

## 2. Methods

Several databases including PubMed, Embase, and Cochrane Library were searched comprehensively, focusing on studies published between 2014 and 2024 that examined the correlation between hs-cTnT, NT-proBNP, and death/cardiac-related events. Data was extracted and pooled using meta-analytic techniques to estimate the pooled values.

### 2.1. Study Design

This systematic review and meta-analysis was done using the Preferred Reporting Items for Systematic Reviews and Meta-Analyses (PRISMA) guidelines. The aim was to evaluate the independent predictive value of NT-proBNP and hs-TnT for cardiac-related events and/or survival in adult patients with CHD over the past decade (2014–2024).

### 2.2. Literature Search

Several electronic databases, including PubMed, Embase, and Cochrane Library were searched using the following keywords and MeSH terms: “N-terminal pro-B-type natriuretic peptide,” “cardiac events,” “congenital heart disease,” “survival,” “high-sensitive troponin T,” and “predictive value.” The search was restricted to English studies published between January 1, 2014, and July 31, 2024. Additional manual search was conducted through reviewing the references of the relevant papers.

### 2.3. Inclusion and Exclusion Criteria

Studies were eligible for inclusion if they focused on adults (aged 18 years and older) diagnosed with CHD and involved the NT-proBNP and hs-TnT levels. Eligible studies needed to report on survival rates and/or the incidence of cardiac-related events. Cohort studies, randomized controlled trials, and case-control studies published between 2014 and 2024 were included. We excluded studies that did not report outcomes related to survival or cardiac events, those involving pediatric populations (under 18 years), as well as case reports, reviews, editorials, and studies with insufficient data for extraction. In cases where there was more than one article extracted from the same population, only one of them was included, which provided the most accurate and relevant data to this study.

### 2.4. Data Extraction and Quality Assessment

The titles and abstracts were firstly screened by two independent reviewers (S.R. and Z.M.), and they initially screened titles and abstracts to determine eligibility. Full texts of studies deemed potentially eligible were then retrieved for further assessment. Any discrepancies between the reviewers regarding study inclusion were resolved through discussion, with a third reviewer consulted if necessary.

The following data were extracted from the included studies: study characteristics (including author, year, study design, and sample size), participant characteristics (such as age, sex, and body mass index (BMI)), biomarker levels (NT-proBNP and hs-TnT), and key outcomes (including survival rates and the incidence of cardiac-related events). Additionally, effect sizes (expressed as hazard ratios (HRs)), along with their 95% confidence intervals (CIs) and *p* values, were recorded.

### 2.5. Statistical Analysis

Pooled HRs and their corresponding 95% CIs were calculated using either a fixed-effects or random-effects model, depending on the degree of heterogeneity across studies. Heterogeneity was assessed using the *I*^2^ statistic, with values above 50% indicating substantial heterogeneity. For each biomarker, both crude and adjusted hazard ratios (aHRs) were analyzed separately. Although the covariates included in the multivariable models varied across studies, aHRs were pooled to capture overall trends while acknowledging potential model heterogeneity.

Publication bias was evaluated through visual inspection of funnel plots and quantitatively using Egger's test. A *p* value < 0.05 was considered statistically significant.

To explore the sources of heterogeneity, predefined subgroup analyses were conducted based on differences in study design, patient population, biomarker measurement units, and cut-off values. Furthermore, sensitivity analyses were performed by excluding one study at a time (leave-one-out method) and by conducting focused reanalyses that excluded specific studies with outlying methodological features (e.g., nontransformed biomarker values or substantially different cut-offs) to assess the robustness and consistency of the pooled estimates. All statistical analyses were performed using STATA.

## 3. Results

### 3.1. Study Inclusion

Two authors (S.R. and Z.M.) independently conducted a search following a specific strategy. A total of 325 records were identified through electronic database searches (EMBASE: 70, ScienceDirect: 60, PubMed: 80, Scopus: 50, Web of Science: 30, and Google Scholar: 30), along with five additional articles identified through manual reference screening. After removing 173 duplicates, 152 unique records remained for screening. Based on titles and abstracts, 137 studies were excluded for being review articles, involving populations outside the scope of this review, being letters to the editor, or lacking full-text access.

**Figure 1:**
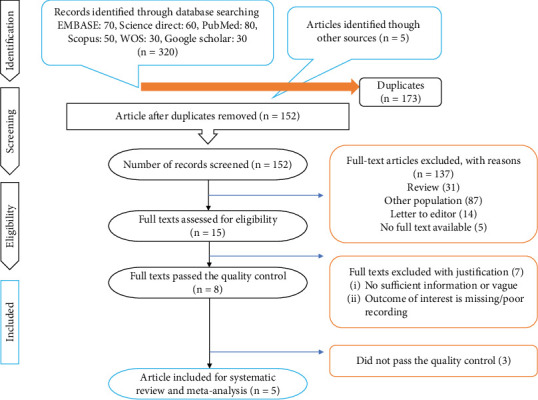
PRISMA flow diagram showing the study inclusion process.

The remaining 15 full-text articles were reviewed in detail. Of these, 7 were excluded due to insufficient reporting, missing outcome data, or methodological limitations. This group also included two studies that used overlapping patient populations already represented in the study by Baggen et al., which was retained for meta-analysis due to its more comprehensive dataset. An additional three studies did not meet predefined quality criteria. In the end, five unique studies met all inclusion and quality standards and were included in the systematic review and meta-analysis (Figure 1).

A total of five studies including 1294 ACHD patients were included in this meta-analysis. The studies of Baggen et al. [20], Hendriks et al. [21], and Geneen et al. [23] used the same population, so only Baggen et al.'s study was included in the meta-analysis. Meanwhile, the other two studies were included in our systematic review. The general characteristics of the included studies can be found in Table 1. All of the included studies were prospective cohorts and reported on death and/or cardiovascular events. The mean BMI and age were 24.5(95% CI: 27.1–27.5) and 37.5 years (95% CI: 36.9–38.1), respectively (Table 1).

### 3.2. Meta-Analysis of the HR of Increased NT-proBNP for Cardiac-Related Events and/or Death

**Figure 2:**
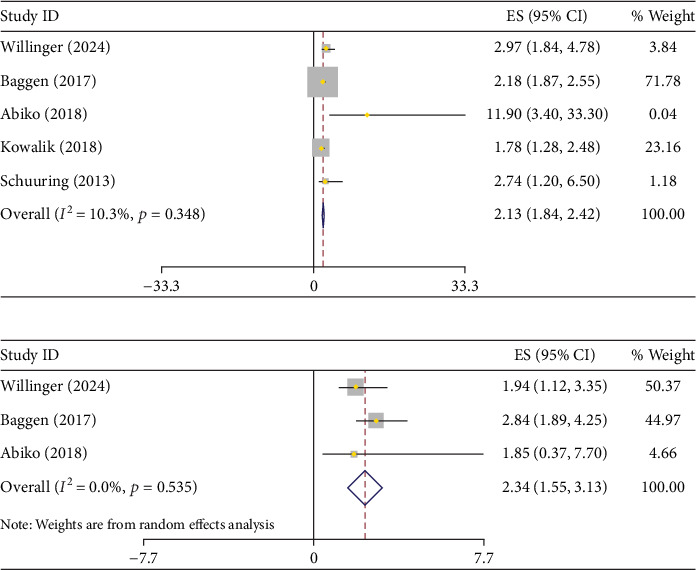
(a) Pulled crude and (b) adjusted hazard ratio of increased NT-proBNP for death or cardiac-related events.

Figure 2 presents the correlation between high NT-proBNP level and the risk of death and/o cardiac events in ACHDs. Overall, adults with CHD who had higher NT-proBNP levels were found to have a significantly increased risk of mortality compared to those with lower levels (HR: 2.13; 95% CI: 1.84–2.42, (*I*^2^ = 10.3%, *p* value = 0.35)).

Additionally, after adjusting for potential confounders, the HR for mortality in patients with elevated NT-proBNP levels remained significantly higher compared to those with lower levels (adjusted HR = 2.34; 95% CI: 1.55–3.13) along with a low heterogeneity (*I*^2^ = 0.0%, *p* value = 0.53). (Table 2).

### 3.3. Meta-Analysis of the HR of Increased hs-TnT for Cardiac-Related Events and/or Death

**Figure 3:**
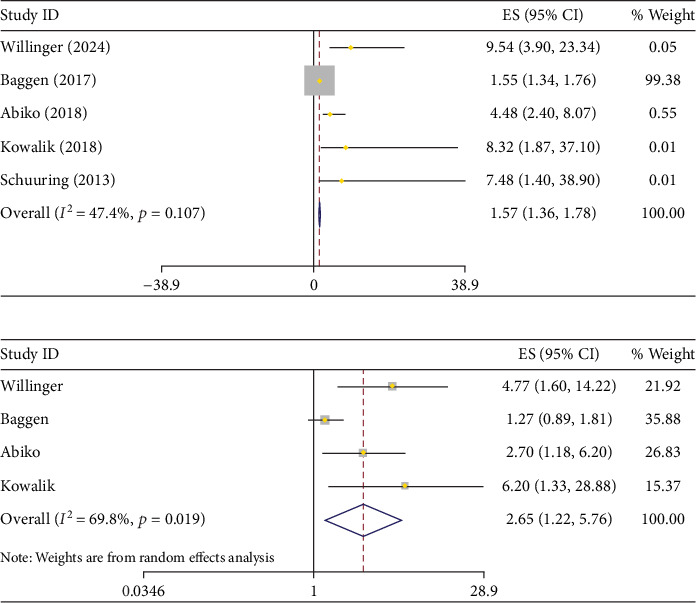
(a) Pulled crude and (b) adjusted hazard ratio of increased hs-TnT for death or cardiac-related events.

Elevated hs-TnT levels were associated with a significantly greater risk of mortality in patients with ACHD. As seen in the data, patients with higher hs-TnT levels demonstrated a marked increase (HR: 1.57; 95% CI: 1.36–1.78, (*I*^2^ = 47.7%, *p* value = 0.11)) in risk of death or cardiac events compared to those with lower levels. The pooled HR for hs-TnT confirmed this heightened risk, showing a statistically significant association, even after adjusting for potential confounding factors (adjusted HR = 2.65; 95% CI: 1.22–5.76, (*I*^2^ = 69.8%, *p* value = 0.02)). (Figure 3 and Table 2).

**Figure 4:**
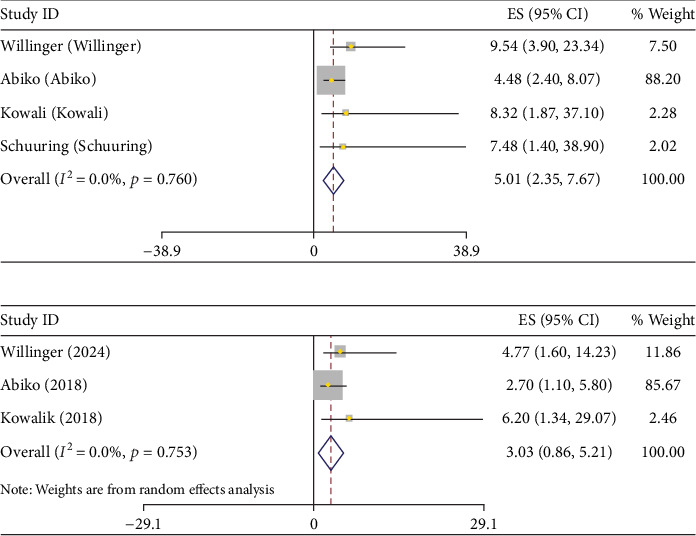
(a) Pulled crude and (b) adjusted the hazard ratio of increased hs-TnT for death or cardiac-related events after sensitivity analysis.

Given the substantial heterogeneity observed in the overall analysis of hs-TnT, a sensitivity analysis was conducted to explore potential sources of variation. Specifically, the study by Baggen et al. [20] was excluded from a subgroup meta-analysis due to its notably lower hs-TnT cut-off value (7.7 ng/L), compared to the other included studies which utilized thresholds of 9 ng/L (one study) and 14 ng/L (three studies). All studies dichotomized hs-TnT as a binary variable, and the variation in cut-off points likely contributed to the observed heterogeneity. After excluding Baggen et al., the pooled crude HR for hs-TnT was 5.01(95% CI: 2.35–7.67, (*I*^2^ = 0.0%, *p* value = 0.76)), and the aHR was 3.03 (95% CI:0.86–5.21, (*I*^2^ = 0.0%, *p* value = 0.75)), both showing strengthened associations with reduced heterogeneity. (Figure 4 and Table 2).

### 3.4. The HR of Increased hs-TnT and NT-proBNP for Cardiac-Related Events and/or Death Based on Countries

**Figure 5:**
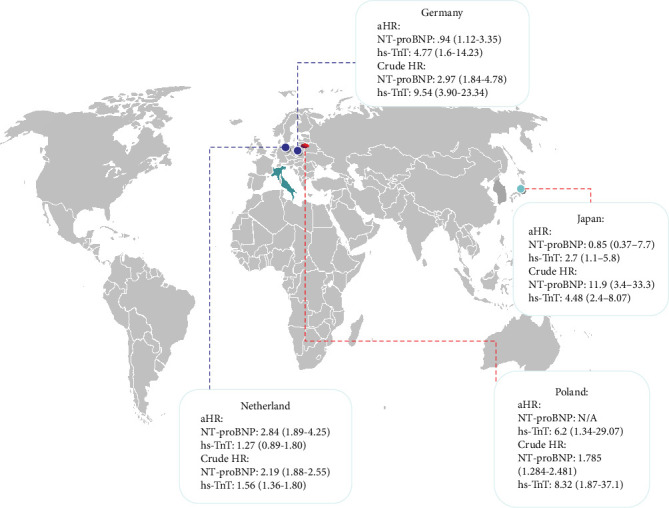
Pulled aHR and HR of increased NT-proBNP and hs-TnT for death or cardiac-related events per country.

Across the included studies, HRs varied by country. In the Netherlands, NT-proBNP showed a stronger association with adverse outcomes (aHR: 2.84; 95% CI: 1.89–4.25) compared to hs-TnT (aHR: 1.27; 95% CI: 0.89–1.80). In Germany, hs-TnT exhibited a higher aHR (4.77; 95% CI: 1.6–14.23) than NT-proBNP (aHR: 0.94; 95% CI: 1.12–3.35). In Japan, hs-TnT had an aHR of 2.7 (95% CI: 1.1–5.8), while NT-proBNP showed a weaker association (aHR: 0.85; 95% CI: 0.37–7.7). Poland reported an aHR of 6.2 (95% CI: 1.34–29.07) for hs-TnT; aHR for NT-proBNP was not available (Figure 5).

## 4. Discussion

The current systematic review evaluated the prognostic potential of hs-TnT and NT-proBNP for death/cardiac events in ACHD cases. High levels of both NT-proBNP and hs-TnT were shown to be predictors of cardiac events/death among adult patients with CHD. Although hs-TnT showed a higher HR, this difference should be interpreted with caution, as NT-proBNP was typically analyzed as a continuous variable, while hs-TnT was assessed dichotomously. As such, the two cannot be directly compared and no conclusion can be drawn about which is the superior prognostic marker.

Persistently elevated blood biomarkers, even in the absence of symptoms, hold clinical significance in managing stable ACHD patients. Regular monitoring of these biomarkers can help in risk assessment and early identification of subtle cardiac decline. [19]

Our study showed that NT-proBNP can act as a strong predictor of death and cardiac events in ACHD patients, with a pooled aHR of 2.34 (95% CI: 1.55–3.13). The prognostic value of NT-proBNP extends even to clinically stable individuals, which can be the result of ongoing myocardial stress [20, 26]. NT-proBNP secretion increases significantly in response to elevated intracardiac pressures, ventricular dilation, and chronic ventricular overload—common pathophysiological conditions in ACHD patients [27–29]. These findings align with previous studies reporting NT-proBNP as a key marker of adverse outcomes and mortality in ACHD during short-term follow-up [20, 30, 31].

On the other hand, our study showed an aHR of 2.65 (95% CI: 1.22–5.76) for hs-TnT in predicting death and cardiac events, which increased to 3.03 (95% CI: 0.86–5.21) in the sensitivity analysis. Elevated hs-TnT serves as an indicator of cardiomyocyte injury, and rising hs-TnT levels over time may suggest ongoing release from the myocardium due to wall strain [23], indicating subclinical myocardial damage. In addition to cardiomyocyte injury, hs-TnT release may also result from inflammation or myocardial/subendocardial ischemia, which could explain its prognostic significance for adverse cardiovascular events, including heart failure [32].

Geneen et al., in their study, evaluating the prognostic value of single and serial measurements of hs-TnT levels in ACHD patients, indicated that individuals with undetectable hs-TnT levels had a notably low risk of adverse cardiac events. The absence of troponin release may, therefore, signal a lack of ongoing myocardial injury or stress, helping to identify patients at low risk. For identifying high-risk patients, however, the authors recommended using hs-TnT in combination with NT-proBNP rather than as a standalone marker. They proposed that if cardiomyocyte loss results from elevated wall stress associated with heart failure progression [23], then NT-proBNP (which is released in response to increased wall stress) [23] would rise before hs-TnT levels increase. This sequence may explain why NT-proBNP retained prognostic value independent of hs-TnT in their findings, as well as in one of the studies [20] included in our meta-analysis. Additionally, the same reasoning could partly clarify the higher aHR for hs-TnT reported by Kowalik et al. [24], which did not account for NT-proBNP levels in their analysis.

Our study showed higher HR and aHR for hs-TnT after sensitivity analysis compared to NT-proBNP in predicting cardiac events/death. Meanwhile, these results should be interpreted carefully since NT-proBNP was typically analyzed as a transformed continuous variable, while hs-TnT was assessed dichotomously. Therefore, these two cannot be directly compared, and no conclusion can be drawn about which is the superior prognostic marker. Meanwhile, research has indicated that measuring both hs-TnT and NT-proBNP together provides a more accurate prediction of survival outcomes compared to measuring hs-TnT alone [25]. Willinger et al. in their recent study reported that patients with elevated levels of one biomarker had a 3.3-fold higher risk for death and cardiac-related events compared to those without increased biomarkers. Meanwhile, if the patients had elevated levels of both biomarkers, they would show a 7.7-fold higher risk for death and cardiac-related events compared to those without increased biomarkers [19].

hs-TnT and NT-proBNP tests are cost-effective, readily available, and provide analytically reliable and accurate results [24]. One strategy can be to use hs-TnT as a particularly useful biomarker to identify low-risk patients. However, when assessing outcomes in higher-risk individuals, a combined approach using both hs-TnT and NT-proBNP may offer greater prognostic accuracy and clinical utility.

This review has several limitations that need to be considered. There was marked heterogeneity across the included studies, including differences in study design, biomarker cut-off values, patient populations, follow-up durations, and statistical approaches used to derive aHR. Specifically, studies varied in the selection and number of covariates included in multivariable models, which may have influenced the comparability and interpretation of the adjusted estimates. Some studies also had relatively small sample sizes or limited follow-up periods, potentially affecting the stability and precision of the HR estimates. Furthermore, due to insufficient disease-specific data, we were unable to perform subgroup analyses based on underlying congenital heart defects.

Future studies should be aimed at comparing hs-TnT and NT-proBNP using consistent methodologies, ideally analyzing both biomarkers as continuous variables and establishing clinically meaningful cut-off values. This would allow for more reliable comparisons and a clearer understanding of their relative prognostic value. It is also suggested to consider the cost-effectiveness analyses to evaluate the economic viability of routine biomarker monitoring programs.

## 5. Conclusion

This meta-analysis shows the prognostic value of both NT-proBNP and hs-TnT in adults with CHD. Each of these markers offered a distinct but complementary clinical insight. Although methodological differences of the included studies limit direct comparison, our systematic review supports the potential value of incorporating both biomarkers into routine risk assessment.

## Figures and Tables

**Table 1:** General characteristics of the included studies.

**Author (year)**	**Country**	**Duration**	**Study design**	**Age**	**Population**	**BMI**	**Male %**	**Sample size**	**Biomarkers measured**	**Key outcomes**	**Follow**	**Death**
Willinger (2024) [19]	Germany	February 2015–October 2019	Prospective cohort	43.9 ± 10	Adults with CHD	25.09 ± 4.	243 (50.9)	495	Hs-TnT, NT-proBNP	Subclinical hs-TnT and NT-proBNP levels are reliable markers for predicting adverse cardiac outcomes and survival in stable ACHD patients.	2.8 ± 1.0 years	53 (10.7%)
Baggen (2017) [20]	Netherlands	April 2011 and April 2013	Prospective cohort	33 (25–41)	Adults with CHD	24.7 ± 4.4	100%	595 (346 (58%male)	NT-proBNP, hs-TnT, and GDF-15	NT-proBNP enhances risk prediction in ACHD, accurately identifying low-risk patients with high negative predictive value.	At the end of the follow-up (August 1, 2015)	12
Hendriks (2024) [21]	Netherlands		Prospective cohort	32.5 (24.7–41.2)	Adults with CHD	24.2 (21.7–27)	58%	602	(NT-proBNP), high-sensitive-troponin T, high-sensitive-C-reactive protein, full blood count, growth differentiation factor 15, renal function, HDL and LDL	Blood biomarkers hold prognostic significance in adult CHD, with NT-proBNP enhancing risk assessment and identifying low-risk patients. Routine use in adult CHD care is recommended.	10.1 years (IQR: 9.7–10.5),	41 (6.8%)
Abiko (2018) [22]	Japan	June 2011–March 2013	Prospective cohort	32 (25.43)	CHD patients	NA	51.6%	122	Hs-TnT, NT-proBNP	High levels of hs-cTnT are reliable indicators of cardiovascular events and cardiac failure severity, making it a valuable prognostic tool for patients with CHD.	3 years	6
Geneen (2020) [23]	Netherlands	2011–2013	Prospective cohort	32.5	Adults with CHD	24.7	NA	601	Hs-TnT, NT-proBNP	In stable ACHD patients, hs-TnT levels rose prior to events, and repeated measurements were linked to the risk of adverse cardiac outcomes. However, repeated hs-TnT did not outperform repeated NT-proBNP.	5.8 years	NA
Kowalik (2018) [24]	Poland	January 2012 –December 2014	Prospective cohort	36 (14.2)	Adults with congenitally corrected transposition of the great arteries	NA	59%	51	Hs-TnT, NT-proBNP	Measurable levels of high-sensitivity troponin T together with an enlarged systemic right ventricular end-diastolic area (≥ 26.4 cm^2^) from echocardiography most accurately predict adverse clinical outcomes.	3.15 years	2
Schuuring (2013) [25]	Netherlands	January 2005–March 2007	Prospective cohort	45 (12)	Adults with pulmonary arterial hypertension due to CHD	Na	39%	31	Hs-TnT, NT-proBNP	Measurable levels of high-sensitivity troponin T were inversely correlated with survival.	5.6 years	8

**Table 2:** Meta-analysis of the hazard ratios of high NT-proBNP and hs-TnT levels for death or cardiac-related events among ACHD patients.

**Study (author, year)**	**AHR**		**Crude HR**		**Cut-off**		**Adjusted for**	**End point**
	**NT-proBNP**	**Hs-TnT**	**NT-proBNP**	**Hs-TnT**	**NT-proBNP**	**Hs-TnT**		
Willinger (2024) [19]	1.94 (1.12–3.35	4.77 (1.6–14.23)	2.97 (1.84–4.78)	9.54 (3.90–23.34)	200 ng/L	> 9 ng/L	High-sensitive troponin T, NT-proBNP, CRP,	Death or cardiac-related events
Baggen (2017) [20]	2.84 (1.89–4.25)	1.27 (0.89–1.80)	2.18 (1.87–2.55)	1.55 (1.34–1.78)	> 33.3 pmol/L	> 7.7 ng/L	Congenital diagnosis, sex, age, systemic ventricular function (0–3), age at surgical repair, NYHA Classes II–III, cardiac medication use, sinus rhythm, saturation < 90%, LA volume, LV end-systolic volume, *E*′ wave, and thickness of the interventricular septum.	Cardiovascular event
Abiko (2018) [22]	1.85 (0.37–7.7)	2.7 (1.1–5.8)	11.9 (3.4–33.3)	4.48 (2.4–8.07)	NA	> 14 ng/L	BNP, age, Cre, FS, EL group, and NYHA Class III.	Cardiac-related events
Kowalik (2018) [24]	N/A	6.2 (1.34–29.07)	1.785 (1.28–2.48)	8.32 (1.87–37.1)	N/A	> 14 ng/L	NYHA, corrected QT interval, systemic right ventricular end-diastolic area	Death or cardiac-related events
Schuuring (2013) [25]	N/A	N/A	2.74 (1.20–6.50)	7.48 (1.4–38.9)	> 49.4 pmol/L	> 14 ng/L	N/A	Death
Pulled value	2.34 (1.55–3.11)	2.65 (1.22–5.76)	2.13 (1.84,2.42)	1.57 (1.36–1.78)	N/A	N/A	N/A	Death or cardiac-related events
Pulled value after sensitivity analysis	N/A	3.03 (0.86–5.21)	2.13 (1.84–2.41)	5.01(2.35–7.67)	N/A	> 14 ng/L and > 9 ng/L	N/A	Death or cardiac-related events

## Data Availability

The data that support the findings of this study are available from the corresponding author upon reasonable request.
